# Direct fluorescence imaging of lignocellulosic and suberized cell walls in roots and stems

**DOI:** 10.1093/aobpla/plaa032

**Published:** 2020-06-29

**Authors:** Peter Kitin, Satoshi Nakaba, Christopher G Hunt, Sierin Lim, Ryo Funada

**Affiliations:** 1 School of Environmental and Forest Sciences, University of Washington, Seattle, WA, USA; 2 Institute of Global Innovation Research, Tokyo University of Agriculture and Technology, Fuchu-Tokyo, Japan; 3 Faculty of Agriculture, Tokyo University of Agriculture and Technology, Fuchu-Tokyo, Japan; 4 USDA Forest Products Laboratory, Madison, WI, USA; 5 School of Chemical and Biomedical Engineering, Nanyang Technological University, Singapore, Singapore

**Keywords:** 3D imaging, autofluorescence, cellulosic wall, Congo red, fluorol yellow, glycerol clearing, histochemistry, lignin, lipids, suberin

## Abstract

Investigating plant structure is fundamental in botanical science and provides crucial knowledge for the theories of plant evolution, ecophysiology and for the biotechnological practices. Modern plant anatomy often targets the formation, localization and characterization of cellulosic, lignified or suberized cell walls. While classical methods developed in the 1960s are still popular, recent innovations in tissue preparation, fluorescence staining and microscopy equipment offer advantages to the traditional practices for investigation of the complex lignocellulosic walls. Our goal is to enhance the productivity and quality of microscopy work by focusing on quick and cost-effective preparation of thick sections or plant specimen surfaces and efficient use of direct fluorescent stains. We discuss popular histochemical microscopy techniques for visualization of cell walls, such as autofluorescence or staining with calcofluor, Congo red (CR), fluorol yellow (FY) and safranin, and provide detailed descriptions of our own approaches and protocols. Autofluorescence of lignin in combination with CR and FY staining can clearly differentiate between lignified, suberized and unlignified cell walls in root and stem tissues. Glycerol can serve as an effective clearing medium as well as the carrier of FY for staining of suberin and lipids allowing for observation of thick histological preparations. Three-dimensional (3D) imaging of all cell types together with chemical information by wide-field fluorescence or confocal laser scanning microscopy (CLSM) was achieved.

## Teaser

Investigating plant structure is fundamental in botanical science. Across disciplines, such as plant development, ecophysiology and biotechnology, particular interest is focused on the complex structure of lignocellulosic walls. We review the recent innovations in tissue preparation and fluorescence staining for lignin and suberin. We demonstrate simple and cost-effective protocols for preparation of plant samples for microscopy using hand-cut sections and clearing with glycerol. Autofluorescence of lignin in combination with direct stains such as Congo red and fluorol yellow can clearly differentiate between lignified, suberized and unlignified cell wall domains.

## Introduction

A reignited interest in studies of plant structure is evident in the last two–three decades triggered by the crucial economic and ecological importance of plants as renewable sources of energy and biomaterials (Van de Wouwer *et al.* 2018; Ralph *et al.* 2019). The lignified and suberized cell walls have vital physiological functions in structural support, defence and water transport, and no less importantly, constitute a major part of the terrestrial biomass (Baas *et al.* 2004). Anatomical investigations of cell wall structure play essential roles in disciplines such as plant ecophysiology, taxonomy, plant development and plant biomechanics (Carlquist 2001; Hacke *et al.* 2006; Rossi *et al.* 2012; Beeckman 2016; Deslauriers *et al.* 2016; Özparpucu *et al.* 2017; Begum *et al.* 2018). In modern studies, the formation and characterization of lignocellulosic or suberized cells are frequently targeted (Abe *et al.* 1997; Chaffey 2002; Chaffey *et al.* 2002; Salmén and Burgert 2009; Yamagishi *et al.* 2012; Pesquet *et al.* 2013; Tobimatsu *et al.* 2013; Kudo *et al.* 2014, 2018; Daniel 2016; Chabbert *et al.* 2018; Abbas *et al.* 2019). However, often the applied protocols for plant sample preparation are decades old and appear to be trailing the rapid progress in light microscopy equipment. It is not surprising that classical methods of tissue processing and microscopy, such as the preparation of paraffin- or plastic-embedded material and semi-thin sections followed by conventional light microscopy, remain in demand because they are proven to be robust and effective. At the same time, innovative approaches in plant anatomical work are continually being developed, with the aim of easing, speeding up and at the same time improving the quality of microscopic investigations. The innovations include every main step: plant material preparation, such as the fixation and clearing of plant material, the methods of sectioning and staining, as well as exciting new developments of the microscopy equipment (Kitin *et al.* 2003, 2010; Lux *et al.* 2005; Sano *et al.* 2005; Truernit *et al.* 2008; Liesche *et al.* 2013; Thomas *et al.* 2013; Palmer *et al.* 2015; Hasegawa *et al.* 2016; Ursache *et al.* 2018; Yazaki *et al.* 2019).

Wide-field fluorescence and confocal laser scanning microscopy (CLSM) are commonly used microscopy techniques in modern biological research (Hepler and Gunning 1998; Blancaflor and Gilroy 2000; Pawley 2006). The principles of fluorescence staining of plant cell walls with a wide variety of fluorescent techniques and applications are reviewed by Running and Meyerowitz (1996), Donaldson and Bond (2005), Drnovšek and Perdih (2005), Paës (2014), Nakaba *et al.* (2015), Yeung *et al.* (2015), Hubbe *et al.* (2019). Compared with conventional light microscopy, fluorescence microscopy offers some advantages that are particularly relevant to applications in plant anatomy. For instance, the clarity of images by epifluorescence depends on the sample surface preparation and less on the thickness of section which allows surfaces to be observed with a high-resolution detail. The possibility to observe thick sections is particularly useful with plant samples where tissues are highly anisotropic and understanding the interrelationships between cells requires large samples and three-dimensional (3D) information. Previously, 3D reconstructions of plant vasculature have been performed using series of mechanical sections and conventional or video light microscopy (Zimmermann and Tomlinson 1966; Tomlinson *et al.* 2001). In addition, X-ray computed tomography (Steppe *et al.* 2004; Trtik *et al.* 2007; Brodersen 2013), as well as tomography by electron microscopy in the nanoscale (Sarkar *et al.* 2014) were applied in 3D studies of biological samples. By microcasting and scanning electron microscopy (SEM), xylem vessel network together with submicron features of cell walls can be visualized in 3D (Kitin *et al.* 2001, 2004). Also 3D reconstructions of plant cells were performed using series of thick mechanical sections and wide-field fluorescence microscopy, or optical sections by CLSM (Bougourd *et al.* 2000; Kitin *et al.* 2000, 2002, 2009; Funada 2002; Haseloff 2003; Truernit *et al.* 2008; Yahya *et al.* 2011). Fluorescence CLSM allows 3D analyses of the cellular structure of large cells with superior lateral (x and y) and vertical (z) resolution (Gray *et al.* 1999; Kitin *et al.* 2002, 2003). In addition, fluorescence staining is very sensitive to the cell molecular structure and can yield higher specificity and contrast, such as information on the lignocellulosic wall composition (Donaldson 2002; Drnovšek and Perdih 2005; Donaldson *et al.* 2010; Donaldson and Radotic 2013; Houtman *et al.* 2016). Furthermore, fluorescence depends on the chemical nature of the molecular environment providing information such as acidity or lipophilicity. Quantitative microscopy based on fluorescence remains a challenge, however, because it is not easy to control the chemical environment.

Many previously published studies on traditional stains either targeted particular cellular components, such as lignin or suberin domains (Brundrett *et al.* 1988, 1991; Lux *et al.* 2005, 2015; Ursache *et al.* 2018), or the 3D cellular structure of tissues and organs with little topochemical detail (Bougourd *et al.* 2000; Kitin *et al.* 2002, 2003; Dubrovsky *et al.* 2006; Coiro and Truernit 2017; Tofanelli *et al.* 2019). Our goal is to present an integrative approach of both visualizing the tissue structure and the cellular chemistry while utilizing the advantages of fluorescence microscopy in terms of rapid and effective sample preparation and observation. Our focus is on the extremely diverse and at the same time intricate structure of cortical and vascular tissues of plants exhibiting secondary growth. We discuss popular fluorescence microscopy techniques with emphasis on lignocellulosic and suberized cell walls and present detailed descriptions of our own approaches and protocols. We present examples of the anatomy of roots and stems of some woody plants including mangroves. Although our discussion is focused on root and stem tissues, the preparation and staining protocols are applicable to suberized, lignified and cellulosic cell walls in other plant organs such as leaves, fruits or seeds.

### The importance of plant material preparation for successful imaging

State-of-the-art microscopy is often perceived as using the latest equipment and fluorescence-labelling techniques which by itself should guarantee superior microscopic images. However, the success of microscopy observations is heavily dependent on the quality of the study material, i.e. appropriately selected and adequately processed for the particular observation samples. Epifluorescence microscopy of thick sections is associated with relatively quick and non-cumbersome sample preparation. Yet, the quality of sample preparation is crucial, particularly with plant material collected after secondary growth because the tissues are mixtures of cell types with contrasting mechanical and optical properties. Plant cell walls (both primary and secondary) serve like shells covering the living content of the cell and give the cell’s particular shape while at the same time performing important functions of mechanical support and protection, while facilitating cell-to-cell transport and communication (Scheres 2001; Funada *et al.* 2001; Chaffey *et al.* 2002; Fukuda 2004; Barnett 2006; Kitin *et al.* 2009; Yamagishi *et al.* 2012). For serving these functions, plant cell walls are typically rigid with complex chemistry and structural design. Some plant cell types with water-transport or mechanical functions have extremely thick and lignified walls that are strongly fluorescent (Angeles *et al.* 2004). The natural fluorescence can be utilized for direct imaging of cell walls and some metabolites. However, high-quality microscopic imaging of plant specimens often requires ‘optical clearing’ that may involve removal of pigments or other extractives as well as infiltration with a high-refractive index medium. Optical clearing methods have been developed by many researchers (Gardner 1975; Bougourd *et al.* 2000; Warner *et al.* 2014; Kurihara *et al.* 2015; Lux *et al.* 2015; Palmer *et al.* 2015; Hasegawa *et al.* 2016; Musielak *et al.* 2016; Timmers 2016; Tofanelli *et al.* 2019), as well as in our laboratory. Thus, the preparation techniques and the observation by microscopy of plant tissues can be diverse and very different from the microscopy of mammalian cells.

## Materials and Methods

Young root and stem segments, after secondary growth had occurred, were obtained from trees of *Bruguiera gymnorrhiza*, *Picea glauca*, *Pinus ponderosa*, *Populus tremuloides* and *Rhizophora apiculata*. Thick sections (at least 40-µm-thick) or planed specimen surfaces were prepared with a microtome or by freehand using a sharp razor blade or a disposable microtome knife. The sections were observed without staining or after staining with safranin, calcofluor white M2R, Congo red (CR), fluorol yellow 088 (FY), acridine orange (AO) or combinations of safranin/calcofluor, CR/FY, AO/FY. Observations were made by wide-field fluorescence microscopy, or CLSM using ultraviolet (UV) excitation and long-pass (LP) 420 emission, or with standard band-pass (BP) filter sets for DAPI, FITC and Texas Red. Appropriate channels were selected for each of the dyes or for autofluorescence of lignin, suberin and non-structural phenolics. The images obtained by wide-field fluorescence and LP emission filters represent the authentic fluorescence colours. For images obtained with BP emission filters, we merged the blue, green or red fluorescence channels using ImageJ (Rasband 1997–2020) image analysis software. Details and discussions on each of the protocols for sectioning, staining and observation are provided in the following sections.

## Results and Discussion

### Preservation and clearing of plant material

Depending on the purpose of observation, different chemical fixatives have traditionally been used in plant anatomy, such as FAA (ethanol:glacial acetic acid:37 % formaldehyde:water/50:5:10:35); GA (4 % glutaraldehyde solution in 0.1 M phosphate buffer of pH 7.3); 1–3 % OsO_4_; or methanol solutions (Talbot and White 2013). In brief, FAA typically provides adequate fixation of protein in cell walls, nuclei and other cell organelles and has been commonly used in studies by light microscopy of root and stem formation and structure. Glutaraldehyde penetrates more slowly through cell walls compared to FAA, and can be only used for small-size samples, but provides superior fixation of the ultrastructure of cell walls, cytoplasm and cell organelles. Glutaraldehyde and OsO_4_ will cross-link and further stabilize subcellular components and have been used in correlative studies by light and electron microscopy (Kiernan 2000; Watanabe *et al.* 2006). These three chemicals can be applied sequentially on the same plant material, firstly FAA, then GA to provide fixation of relatively large samples, then followed by fixation with OsO_4_ of subsamples for light and electron microscopy (Kitin *et al.* 2002, 2009; Clode 2015). It has to be pointed out that the strong, chemically reactive fixatives are usually toxic if allowed to contact living tissue. Skin or eye contact and inhalation are the most common exposure routes. Therefore, protective wear, a fume-hood and care need to be used when working with such chemicals.

#### Water-ethanol-glycerol solution.

Ethanol or ethanol-glycerol solutions for preservation of plant material are relatively less toxic and easier to handle. Nakaba *et al.* (2015) recommend water/ethanol/glycerol (WEG) mix (in the ratio of 45/45/30 mL) for preservation of cambium and differentiating vascular tissues. In our experience, it is possible to increase the amount of glycerol when effects of plasmolysis do not visually alter the cell morphology and cell wall structure. Immediately after harvesting, the plant sample can be immersed in WEG in equal proportions (1:1:1) which not only preserves the cellular structure but also the glycerol acts as a plasticizer making the suberized and lignified tissues easier to cut. The WEG preservation and storage procedure is easy to do in the lab or in the field. We recommend to vacuum-infiltrate the sample within 2–3 h of harvesting and change to a fresh WEG because extractives from the sample may have entered the storing solution. For long-term storage, we keep the samples refrigerated at 4 °C and refresh the fixative solution at least once a month. Over the course of 2–3 months, we have not observed any microorganism development or degradation of root or stem samples stored in WEG at 4 °C. The structures and chemical content that we have targeted for observation, such as cell wall, nuclei, starch or lipids, have been adequately preserved for light microscopy or SEM observation. The WEG will clear the tissue from extractives, soluble pigments and non-specifically bound dye. The WEG storing solution can be easily replaced with pure glycerol as a mountant for fluorescence observations as further described in our protocols. A popular tissue-clearing agent for microscopy is chloral hydrate; however, it is a regulated drug and might be difficult to obtain. Glycerol as well as glycerol/water solutions, due to high refractive indices and easy penetration into cells, also serve as excellent clearing agents. The refractive indices of aqueous glycerol increase depending on the glycerol concentration as the following, listed by Bochert *et al.* (2005): RI = 1.337 (5 %); 1.355 (20 %); 1.411 (60 %); and 1.470 (100 % glycerol). All micrographs in this paper were obtained from samples that have been either stored in WEG or cryo-fixed as further described.

### Sectioning or surfacing of roots and stems

#### The traditional thin sections of plant material.

Transmitted-light microscopy requires thin histological sections for the light to pass through the sample. Semi-thin sections (1- to 5-µm-thick) can provide high-quality imaging with sharp focus and subcellular detail using the traditional transmitted-light microscopy. Semi-thin sections eliminate the problems of out-of-focus light that causes blur in the images; therefore, semi-thin sections can improve the imaging by both conventional light and fluorescence microscopy (Kitin *et al.* 2000, 2002). Semi-thin histological sections can be prepared from plastic-embedded or paraffin-embedded material using a rotary microtome (O’Brien and McCully 1981; Ruzin 1999; Rahman *et al.* 2019). However, these procedures require several days to be completed. Furthermore, unwanted effects by the dehydration, chemical modification or extraction of soluble substances during the steps of tissue preparation can be expected to occur. An important advantage of the plastic-embedded sections is that fragile cellular structures, such as division plates in cambial cells, pit membranes, perforation partitions of developing vessel members or partially digested or degraded walls, remain intact in their original position. In unembedded sections prepared with a microtome or freehand, thin portions of the cell walls are often torn or displaced. Plastic-embedded sections allow for observation of the morphology and the insoluble content of vacuoles and protoplast. However, it needs to be pointed out that many of the same advantages for preservation and observation of subtle subcellular structures are offered by cryo-sectioning, and to some extent by application of polyethylene glycol (PEG) embedding and preparation of clean-cut surfaces for reflected-light or epifluorescence microscopy as explained below.

#### Thick sections or planed surfaces.

**Figure 1.**
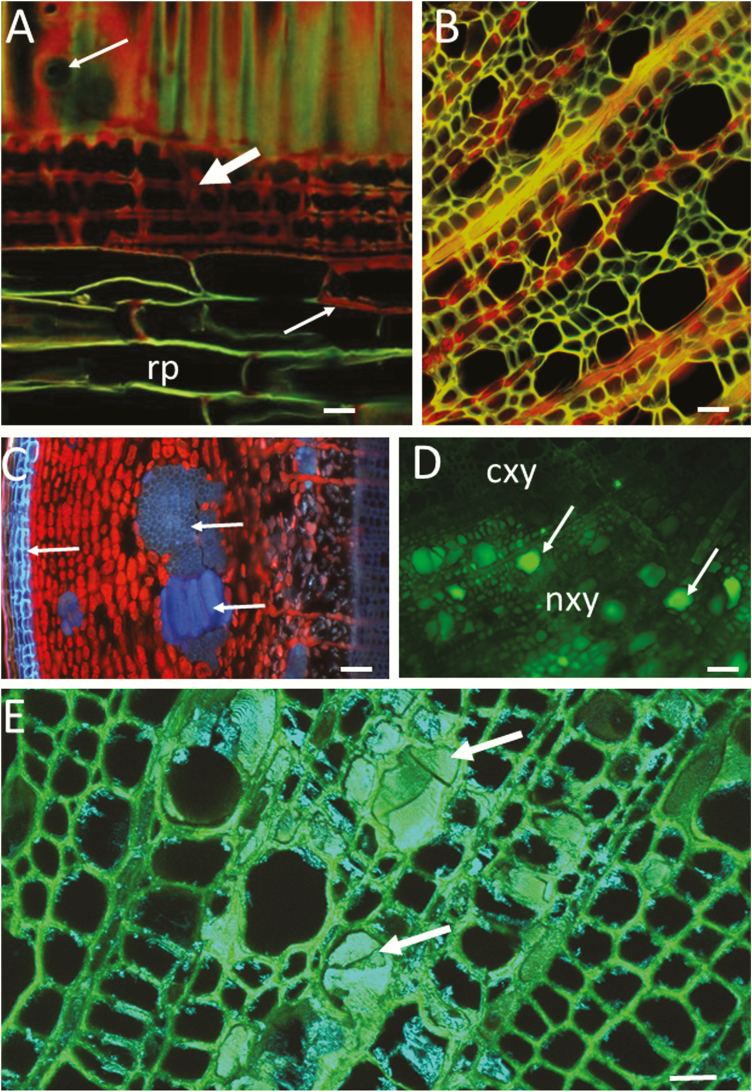
Visualization of lignin, suberin and extractives on microtome-planed surfaces of wood (A, D, E), and on hand-cut sections (B, C). Safranin staining (A) and autofluorescence (B through D). (A) Radial-longitudinal section, ~60-µm-thick, through the sapwood of a ponderosa pine tree (*Pinus ponderosa*) visualized by CLSM after safranin staining. The red colour corresponds to more lignin. The upper arrow points to a bordered pit in the radial wall of an earlywood tracheid. The lignified walls of axial tracheids show a mixture of red and green fluorescence depending on the lignin proportion of the sectioned wall layer, middle lamella (red) or S2 (green). The middle arrow points to ray tracheids, and the lower arrow points to a lignified ray parenchyma cell (indicated with red colour and thick wall). The ray tracheids are strongly lignified (red colour) while most of the ray parenchyma cells in this species have non-lignified walls (a single optical section, Plan-Neofluar 40×/water, merged two-channel excitation\emission: 488\500–530 and 543\LP 590; Zeiss LSM510). (B) Hand-cut, transverse section of a young poplar stem (*Populus* sp.) visualized by wide-field fluorescence (excitation/emission, 450–480/LP 515; Nikon; Eclipse E400). Green is emitted from lignified walls and red from chloroplasts in ray parenchyma. The orange-yellow colour is a mixture of red and green indicating that the horizontal wall of ray cells is near the surface of the section. (C) Transverse cut through the cortex of a 1-year-old poplar stem (*Populus* sp.) after some development of secondary bark has occurred (wide-field with no staining, excitation/emission, 330–385/LP 420; Nikon; Eclipse E400). The uneven focus is due to the rough surface of the sectioned cortex that contains cell types with contrasting density and hardness (see the text for discussion on hand sections). The arrow at the left points to blue fluorescence from cork. The two arrows at the right point to blue fluorescence from lignified sclerenchymatic cells. The red fluorescence is emitted from chloroplasts in cortical parenchyma and rays. A small portion of last-formed xylem (weak, blue fluorescence) can be seen at the right side of the micrograph. (D) Transverse-cut surface of wood of a low-lignin hybrid poplar tree (Kitin *et al.* 2010). The sample was cryo-fixed and cut in the frozen state, then visualized in the frozen state by wide-field fluorescence without staining (blue excitation and band-pass emission BP 510–530; Nikon; Eclipse E400). Xylem areas that are not functional for water conduction (nxy) contain xylem vessels filled with phenolics (arrows) while no occlusions are visible in the conductive xylem (cxy). The lignification of the conductive xylem is normal; however, the lignin autofluorescence is considerably weaker (not visible with the selected laser power/detector gain) compared with the fluorescence of lumen phenolics. (E) A similar wood sample as in (D) but freeze-dried and visualized at room temperature by CLSM (LSM 510; Carl Zeiss). Arrows point to xylem vessels occluded with phenolics. After freeze-drying, the phenolics emit strong blue and green fluorescence. A single-track, two-channel image with 405- and 488-nm laser lines and band-pass filters BP 420–480, BP 505–530. The image is a maximum projection of four optical sections at 1-mm intervals. Bars = 20 µm (A and E), 50 µm (B, C and D). cxy, conductive xylem; nxy, non-conductive xylem; rp, ray parenchyma.

Microscopes with high-quality reflected-light optics or confocal microscopes are becoming more easily available in university campuses or centralized microscopy facilities. With a wide-field fluorescence microscope, superior images can be obtained from the surfaces of samples without the need for thin sections (Figs 1 through 5). Clean and smooth surfaces can be achieved by shaving away very thin layers of the tissue with a sharp knife or a microtome blade (Kitin *et al.* 2010; Yazaki *et al.* 2019). Artificial distortions of shapes or cell wall damage are less common in thick sections than in thin ones. For stabilization of soft tissues such as cambium and phloem during sectioning, some authors employ embedding in PEG as explained further in this paper. Thick sections are suitable for 3D imaging of the morphology and cell wall development of large vascular cells (Kitin *et al.* 2003, 2004, 2016). Making thick sections with a sliding microtome is easy to learn and requires less preparation time compared to sectioning of epoxy-embedded material. Planed surfaces are particularly adequate for plant cryo-microscopy (for a detailed description of the cryo-planing procedure, see Yazaki *et al.* 2019). It is considerably faster to prepare planed surfaces compared to making thin or thick sections.

An interesting technique of polishing the surfaces of epoxy-embedded tissue blocks followed by CLSM is described by Dickson *et al.* (2017). Sanded wood surfaces can be also appropriate for reflected-light microscopy as described by Cerre (2012) and Hunt *et al.* (2018), though lumina between ~5 and ~80 µm get plugged with dust. Tissue blocks or thick sections allow for large-area observations which can be especially useful in studies of roots and stems, and in particular, when we need to scale up from the subcellular to the tissue- or organ-level investigation. For reflected-light or epifluorescence microscopy, the quality of observation depends mainly on the smoothness of the surface of the section and less on its thickness. Light colour is indication of increased surface roughness and light scattering, while surfaces become darker and reflective as they become smoother. For example, a light-coloured sawn surface becomes much darker as damaged cells are polished or cut away.

#### Microtome versus freehand sections.

Microtomes are designed to provide sections with precisely determined and uniform thickness. Vibratomes are helpful for cutting soft plant organs such as leaves, shoots and young roots (Gunawardena *et al.* 2007; Clode 2015). Sliding (sledge) microtomes are the most common instrument in anatomical studies of wood or tree bark and are typically employed for making thick sections in the range from 10 to 60 µm of thickness (Jansen *et al.* 1998; Dié *et al.* 2012; Delvaux *et al.* 2013; Kitin and Funada 2016). Sliding microtomes are great tools for cutting series of sequential sections for 3D reconstructions of plant vasculature (Zimmerman and Tomlinson 1966; Kitin *et al.* 2004). Sliding microtomes have also been successfully employed for planing of wood surfaces for ecological or functional studies of xylem structure (Utsumi *et al.* 2003; Gärtner and Nievergelt 2010; Kitin *et al.* 2010).

Sliding microtomes are suitable for cutting samples with rigid cell walls and relatively uniform hardness such as lignified or suberized tissues. However, making even cuts through tissues that contain high proportions of unlignified cells is problematic, and therefore, embedding of the tissue in media that can stabilize the soft cells is often implemented. Embedding in paraffin or epoxy is associated with the use of rotary microtomes and preparation of ultra-thin or semi-thin sections. For preparation of thick sections with a sliding microtome, embedding in PEG has been successfully employed as explained further in this paper.

Freehand sectioning is faster, cheaper, easy to learn and can provide high-quality sections for light microscopy. Freehand sections have been routinely used in many laboratories in particular for studying roots and leaves or xylem and phloem. A variety of techniques can be employed for cutting plant material by freehand (Ruzin 1999; Wiedenhoeft 2011; Yeung *et al.* 2015). We use a cutting board and a sharp razor blade or scalpel to cut thin slices of roots or stems. Cutting while observing the sample under a dissecting microscope is helpful for controlling the precise position and direction of the cut. Cutting gently with slightly advancing movement of the blade may help achieve fairly thin and flat sections with intact arrangement of cortical and vascular tissues. Cutting small areas reduces the overall cutting force and typically results in better sections. The freehand sectioning can be facilitated by applying a paraffin sheet (Frohlich 1984) or 6 % agarose (Zelko *et al.* 2012) over thin roots to keep them stabilized during cutting. Furthermore, young and unlignified roots or stems can be cut after PEG embedding, as described below, which stabilizes the sample from either outside and inside as low molecular weight PEG easily infiltrates plant tissues. For fully lignified tissue, each section of razor blade can often only make one premium quality cut.

An important note to be made is that before freehand or microtome sectioning, FAA or other fixative media has to be well washed out from the specimens. We recommend rinsing FAA-fixed samples for 15–30 min in running tap water before cutting sections for microscopy. The plant material has to be wetted with water or glycerol before sectioning except for PEG-embedded material (see notes in the following paragraph). Depending on the hardness of the tissue, blades may have to be frequently changed.

#### Embedding in PEG 1500.

Biological sample embedment in PEG (carbowax) for light and electron microscopy has long history in both plant and animal anatomy. Polyethylene glycol is soluble in water and can be easily removed from the sections before staining and observation. For this reason, PEG embedding has been useful for immunostaining or *in situ* hybridization of sections, unlike the difficult to remove impregnation with waxes or resins (Clayton and Alvarez-Buylla 1988). The melting point of the PEG polymer depends on its molecular weight; for instance, PEG 600 melts at 22 °C, PEG 1000 melts at 32 °C, PEG 1500 melts at 45 °C and PEG 6000 at 60 °C. Therefore, samples can be embedded in PEG 1500 at temperatures above 50 °C and then conveniently stored and cut at room temperature. Riopel and Spurr (1962) used a 19:1 mixture of PEG 1540/PEG 4000 for serial sectioning of potato tuber. Ferreira *et al.* (2014) gradually embedded parts of stems, leaves or insect galls in PEG 6000 for sectioning with a microtome. However, for special applications such as immunostaining, there have been concerns about using higher molecular weight PEG because high temperatures may compromise immunoreactivity. Gao (1993) and Hayat (2000) provide discussions of different PEG embedment techniques in various studies. More recently, PEG embedment was applied in studies of xylem and bark development in plant stems (Schmitz *et al.* 2007, 2008; Barbosa *et al.* 2010; Delvaux *et al.* 2013). Before sectioning, Barbosa *et al.* (2010) covered the PEG-embedded material with polystyrene foam solution or adhesive tape to keep fragile sections intact.

As we discuss later, PEG solution has been used as a carrier of the fluorescent stain FY for labelling of root endodermis (Brundrett *et al.* 1991). Ferreira *et al.* (2014) found that binding of PEG 6000 to polyphenols may affect the histochemical tests for tannins and lignin. They suggested fixation with ferrous sulfate and formalin previous to PEG embedding for detection of total phenolics (Ferreira *et al.* 2017). Alternatively, we remove most of the PEG 1500 from the sections by rinsing for a few minutes in warm water or in 50 % ethanol, which serves well our purpose of observation.

We use the following procedure for embedding root and stem specimens in PEG 1500. The chemically fixed specimens are rinsed for 15–30 min in running tap water. Then, the specimens are immersed in a series of increasing concentrations of PEG in distilled water (ethanol can be used as well) heated to 60 °C. For specimens with size ~1 cm^3^, we use the following series: 30 % PEG 1500 for 1 h; 60 % PEG 1500 for 3 h with one change of the PEG solution; 100 % PEG 1500 overnight while the vials with specimens are kept at 60 °C in oven. On the next day, exchange with a new melted 100 % PEG 1500 for 1 h. The time of infiltration with liquid PEG depends on the size and nature of specimens; for example, larger than 1 cm^3^ specimens with suberized tissue may require longer time for successful infiltration, such as 2–3 days. Finally, the preparations can be transferred to embedding moulds at room temperature for curing the PEG 1500 and the sample blocks will be ready for sectioning. Smearing a thin layer of Vaseline on the surface of the moulds beforehand can help to easily separate the blocks from the moulds. Store the embedded material in air-tight containers to prevent rehydration.

#### Cryo-fixation and cryo-sectioning.

**Figure 2.**
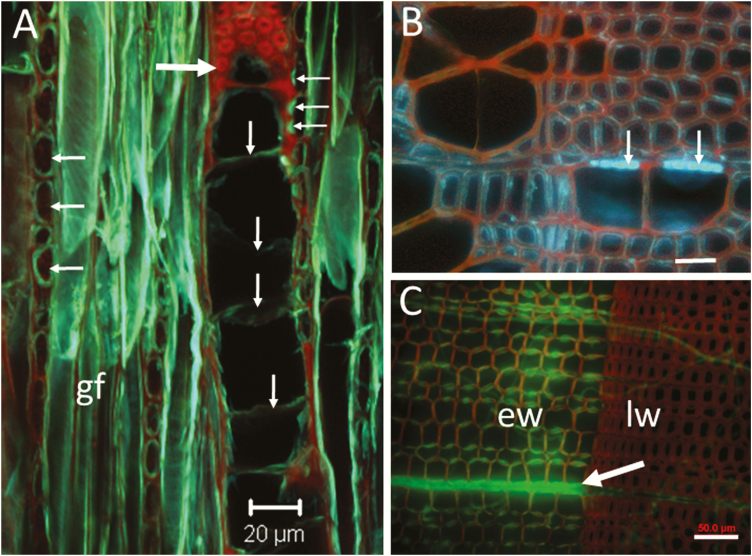
Visualization of lignified and non-lignified cellulosic cell walls in thick sections stained with calcofluor (A) or with safranin-calcolfluor (B, C). The sections are 40- to 60-µm-thick, cut with a sliding microtome. (A) Cellulosic walls (blue-green fluorescence) of gelatinous fibres (gf), ray cells and tyloses in wood of a low lignin poplar mutant. Red autofluorescence is emitted from the lignified vessel wall showing bordered pits. This is a 60-µm-thick tangential-longitudinal section of a sample similar to those in (Fig. 1D and E), but after the extraction of phenolics and stained with calcofluor. Tyloses (vertical arrows); ray cells (horizontal arrows on the left); xylem vessel wall with bordered pits (large arrow); and membranes of parenchyma-to-vessel pits (horizontal arrows on the right) are shown. Image by CLSM (Zeiss, LSM 510; maximum projection of 31 optical sections at 0.95-µm intervals and band-pass filters (Blue: 405 ex/BP 420–480, Green: 488 ex/BP 505–530, Red: 543 ex/BP 590–650)). (B) Transverse section of poplar wood (*Populus tremuloides*) visualized by wide-field fluorescence (Olympus BX60; 330–385 excitation, 420 LP emission). Red fluorescence from lignified walls is visualized simultaneously with blue fluorescence from cellulosic portions of the walls. The arrows point to non-lignified pit membranes of half-bordered pits between ray parenchyma and xylem vessels. Weaker blue signal is emitted from non-pit portions of the lignified walls of fibres where cellulose was exposed to calcofluor staining. The cellulose in lignified walls can become accessible to staining in damaged wall areas such as small cracks that are caused during sectioning. (C) Transverse section of spruce wood (*Picea glauca*) visualized by wide-field fluorescence (Olympus BX60). The section was inoculated with the white rot fungus *Phanerochaete chrysosporium* which removes lignin from cell walls. The arrow points to fluorescence from calcofluor-stained cellulose in a delignified xylem ray 20 days after inoculation. This image was acquired by merging the fluorescence signal from calcofluor-stained cellulose (330–385 excitation, 420 LP emission) with the red fluorescence signal from safranin-stained lignified walls (green excitation, LP 560 emission). Green colour was artificially assigned to the calcofluor signal for a higher contrast (for more details, see the text for discussion on multichannel imaging). The combination of green and red provides a clearer view of the cellulose exposure that occurs predominantly in the rays and in the radial walls (pit regions) of earlywood tracheids. Bars = 20 µm (A) and 50 µm (B and C). ew, earlywood; gf, gelatin fibres; lw, latewood.

Current methods of cryo-microscopy are reviewed in depth elsewhere (Echlin 1992; McDonald and Auer 2006; Kitin *et al.* 2010; Yazaki *et al.* 2019). Cryo-fixation preserves the original cellular structure, water content and position of secondary metabolites or tracer dyes within cell lumens. Frozen specimens can be cut or planed with a cryo-microtome and then observed in the frozen state by cryo-electron microscopy (Utsumi *et al.* 1996, 1999, 2003; Kuroda *et al.* 2009; McCully *et al.* 2009; Yazaki *et al.* 2019), or cryo-light microscopy (Kitin *et al.* 2010; Barnard *et al.* 2013; Vidot *et al.* 2018). Cryotomes for sectioning of frozen samples are particularly useful in studies of soft tissue and these instruments have been often employed in animal research. Cryo-sectioning preserves the subtle cell walls and subcellular structures similarly to the method of cutting plastic-embedded material. Furthermore, rapid-freezing followed by cryo-sectioning preserves the cell water content and distribution of secondary metabolites. Figure 1D shows phenolic deposits in xylem vessels which would have been extracted from the section by conventional sample preparation. Extractives typically cannot be studied in conventional thin sections because they are lost during sectioning and washing of the sample (Kitin *et al.* 2010). Cryo-fixation of extractives as well as sections free from extractives can both provide important information. For example, the location of soluble metabolites is visible in Fig. 1D, but we need to extract them in order to study tyloses, gelatinous fibres and ultrastructural details of lignified and lignin-free cell wall as demonstrated in Fig. 2A. The cryo-fixed samples can be freeze-dried to avoid the inconvenience of maintaining frozen samples. A freeze-dried sample observed with a short-distance objective lens and 3D optical sectioning is shown in Fig. 1E.

The following materials and safety equipment are essential for cryo-fixation and cryo-sectioning: a well-ventilated room and a Dewar for storing liquid nitrogen, a freezer, a method of keeping the sample and knife cold, such as a cryostat or a cold room (walk-in freezer) at −30 °C, sharp knives or disposable stainless microtome blades, long tweezers, gloves and eye protection. More details on different methods of plant cryo-microscopy are provided by Yazaki *et al.* (2019).

### Wide-field fluorescence microscopy and CLSM

Epifluorescence microscopy enables convenient observation of sectioned or planed surfaces of relatively large tissue samples. Furthermore, by fluorescence CLSM, large plant cells, such as xylem vessel elements, or large areas of the tissue can be observed in 3D on both the tissue level and subcellular detail (Kitin *et al.* 2003; Cheng 2006; Nakaba *et al.* 2015). Some fluorescence microscopes can accommodate cryo-stages that allow for observation of samples in the frozen state (Kitin *et al.* 2010). Note that fluorescence spectra can be influenced by molecular environments, such as temperature, pH or concentration and interactions between fluorophores or other chemicals present. Therefore, quantitative fluorescence microscopy is challenging, especially for comparisons across different dates, sites or using different microscopy equipment. Techniques to improve the accuracy and precision of quantitative fluorescence are available (Pawley 2000; Waters 2009).

#### Observation using natural fluorescence (autofluorescence).

Chlorophyll, cutin, suberin and various polyphenols including lignin are naturally fluorescent substances in plant cells (Rost 1995; Hutzler *et al.* 1998; Donaldson *et al.* 2015; García-Plazaola *et al.* 2015; Talamond *et al.* 2015). Autofluorescence (indigenous fluorescence) can be a significant problem when it overlaps with the fluorescence label on structures targeted for observation. A number of techniques are available to suppress the unwanted fluorescence signal. Application of non-fluorescent stain such as bromphenyl blue for quenching the autofluorescence is one example. In certain cases, clear observation of the fluorophore of interest can be achieved by using narrow-band excitation and/or emission filters and adjustments of laser power, or by mathematical spectral unmixing of fluorescence labelling and autofluorescence (Mansfield *et al.* 2005; Zimmermann 2005; Neher *et al.* 2009; Mansfield 2014; Harmany *et al.* 2017). Inspection of the fluorescence excitation and emission spectra of the different parts of the sample can determine whether unmixing is likely to succeed. Another way is to extract the autofluorescing substances by using clearing agents. Dalton *et al.* (2011) extracted the chlorophyll from poplar leaves in order to make visible the fluorescence of polyhydroxybutyrate, a biodegradable plastic that was accumulated in chloroplasts.

On the other hand, autofluorescence can be very useful in plant anatomical studies as it allows fluorescence imaging without staining (Fig. 1B through E) or with combinations of autofluorescence and staining (Figs 2 through 6A and B). For example, different compositions of lignin have different natural fluorescence characteristics and the strength of the fluorescence signal increases with lignin concentration (Albinsson *et al.* 1999; Donaldson 2013). Donaldson (2013) showed that the autofluorescence emission profiles of fibre wall and xylem vessel wall in poplar are different. It was hypothesized that the difference was due to the occurrence of syringyl γ-*p*-hydroxybenzoates in poplar fibre walls but the underlying mechanism remains to be confirmed.

In addition to the typical role of matching index of refraction, mounting media for fluorescence studies should not obscure the fluorescence signal. Donaldson (2013) recommended glycerol at pH 9 as the optimal mounting medium for lignin spectroscopy at the visible excitation range. According to the same author, thiodiethanol, which has a higher refractive index than glycerol, allowed a stronger fluorescence signal as a mounting medium with UV excitation. Strong fluorescence from cytoplasm and primary cell walls can be induced by some fixatives such as glutaraldehyde or WEG. Glutaraldehyde-induced fluorescence was used by Singh *et al.* (1997) to image wood-degrading fungi and by Kitin *et al.* (2000) to image cambial cells. The knowledge of autofluorescence from various plant substances can have important applications in plant histochemical and cell developmental studies; therefore, more investigations of the nature of autofluorescence are desirable. Understanding autofluorescence can be particularly useful for direct observation of frozen plant material after cryo-sectioning.

#### Fluorescence microscopy of lignified and unlignified portions of cell walls.

Advanced techniques such as fluorescence resonance energy transfer (FRET) and fluorescence lifetime imaging (FLIM) have been applied in studies of the interactions of enzymes or fluorophores with complex lignocellulosic walls (Donaldson and Radotic 2013; Houtman *et al.* 2016; Terryn *et al.* 2018). Lignin exhibits autofluorescence over a large spectral range, which has been used for imaging the structure of wood and in studies of delignification or degradation of woody cells (Donaldson *et al.* 1999, 2010; De Micco and Aronne 2007, 2012; De Micco *et al.* 2012; Donaldson 2013). Here, we illustrate an easy way for differentiation between lignified and unlignified cellulosic walls by using the natural fluorescence of lignin in combination with staining of polysaccharides.

#### Congo red staining of cellulose and blue autofluorescence of lignin.

Cellulose is the main building material of plant cell walls and even strongly lignified walls may contain more cellulose than lignin. Between 40 and 50 % of wood consists of cellulose and 20–30 % of hemicelluloses and lignin (Plomion *et al.* 2001; Uraki and Kuroda 2015). For example, the composition of xylem walls of alfalfa has been measured to be 28 % lignin, 4 % pectin, 29 % hemicellulose and 39 % cellulose as compared to 15 % lignin, 25 % pectin, 30 % hemicellulose and 30 % cellulose in lignified non-xylem walls (Grabber *et al.* 2002).

The mechanisms of cellulose staining with different categories of dyes are reviewed by Wallace and Anderson (2012) and Hubbe *et al.* (2019). Traditional stains for cellulosic walls by conventional light microscopy are astra blue, fast green or gentian violet (O’Brien and McCully 1981; Srebotnik and Messner 1994; Ruzin 1999; Chaffey 2002).

Recently, the fluorophores CR and pontamine fast scarlet 4B (PFS) (synonym, direct red 23) have gained popularity because of their affinity to cellulose and chitin (Slifkin and Cumbie 1988; Verbelen and Kerstens 2000; Hoch *et al.* 2005; Anderson *et al.* 2010; Liesche *et al.* 2013; Thomas *et al.* 2013). It is important to keep in mind that most dyes bind by association with their target. Even highly specific protein-binding domains have cross-reactivity. The association of dyes with their targets is usually less specific and so will have even more cross-reactivity. A dye found to be selective in one environment will be non-selective in the presence of a different mounting medium or other chemical binding sites in a different sample. For example, the membrane dye FM 5-95 (Thermo Fisher Cat # T23360) clearly shows fungal membranes and not bleached kraft paper fibres. However, when fibres contain large quantities of oxidized lignin, FM 5-95 is selective for the fibre (Fig. 6C). Another common example is the claim of specific dye affinity to chitin or cellulose. In almost all these cases, the specificity is observed because the samples do not contain both targets.

In our experience, CR (molecular weight 697) is convenient to use because staining is stable in water and glycerol while unbound dye is quickly removed from the sections. The fluorescence of diluted CR in water and alcohol is very weak and practically the microscopic preparations are free from unwanted background fluorescence. The absence of fluorescence contamination is an important advantage particularly for imaging thick sections that are difficult to clear after staining. The excitation/emission maxima of CR in aqueous solution is 502 nm/602 nm (Giri *et al.* 2004), and when bound to cell walls is 561 nm/630 nm (Donaldson and Bond 2005). Therefore, the excitation/emission of CR allows for co-localization studies via multispectral imaging with blue, green and yellow fluorophores, or with autofluorescence as we further demonstrate.

In the 1890s, CR was commonly used to stain cotton but was later abandoned due to toxicity. It is believed that adsorption binds CR to cellulose when its molecules align along the linear molecules of crystalline cellulose (Woodcock *et al.* 1995; Mazeau and Wyszomirski 2012). CR, similar to calcofluor, reportedly has affinity to a larger class of polysaccharides, including glucans, and xyloglucans (Wood *et al.* 1983; Mitra and Loque 2014; see also discussion by Hubbe *et al.* 2019). Congo red stains lipopolysaccharides of Gram-negative bacteria and also has become the classic histochemical probe for amyloids in Alzheimer disease research (Steensma 2001; Yakupova *et al.* 2019). Variations in intensity of CR staining should be considered with caution because of the bifluorescence of this dye, i.e. the fluorescence intensity of CR depends on the orientation of cellulose microfibrils in the image plane. The bifluorescent property of CR has been used for studying the microfibrillar organization of secondary cell wall using polarized optics (Verbelen and Kerstens 2000). Another property of the CR dye that needs to be considered is that under white light illumination its colour changes from blue to red with the increase of the pH from 3.0 to above 5.2 (Steensma 2001). This metachromatic property may offer applications for detection of acidity or for multi-staining. Congo red is possibly carcinogenic and mutagenic and protective wear should be used when working with this dye.

**Figure 3.**
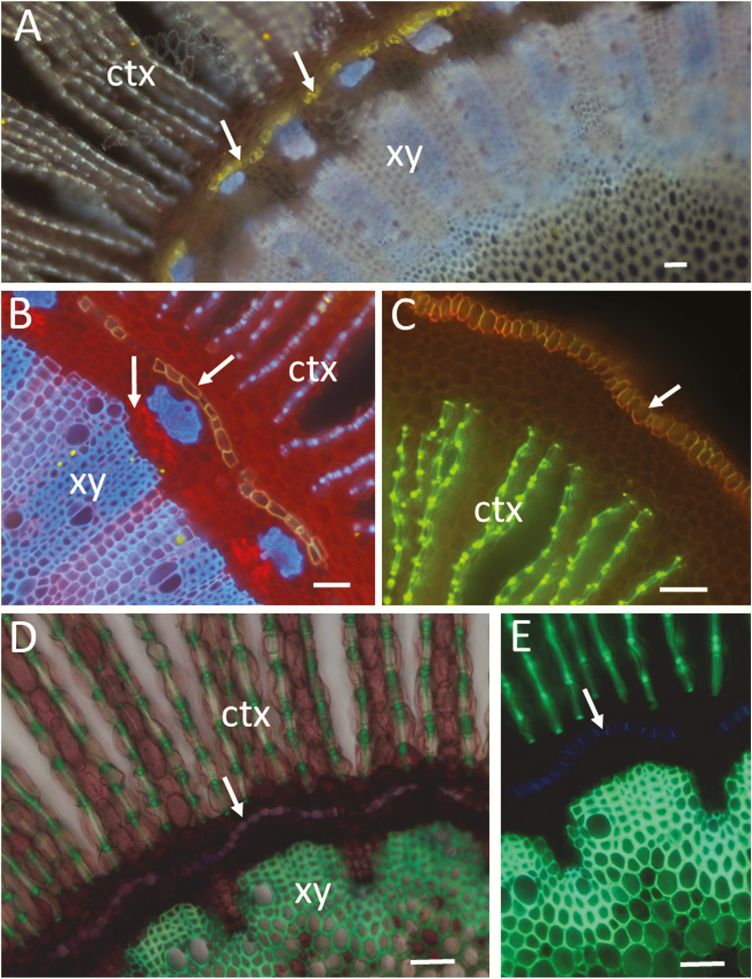
Lignified, suberized and cellulosic walls in hand-cut transverse sections of root of a black mangrove plant (*Bruguiera gymnorrhiza* visualized by wide-field fluorescence (Olympus BX60). (A) Fluorol yellow staining for visualization of suberized endodermis (arrows). Inner cortex (ctx), and xylem (xy) are visualized with the blue autofluorescence of lignified walls (340–380 nm excitation, LP 420 emission). (B) Fluorol yellow/Congo red staining showing suberized endodermis (inclined arrow) and cellulosic walls of cortical and phloem cells (red). Note the stronger red fluorescence from phloem (vertical arrow). Xylem, phloem sclerenchyma and lignified thickenings of cortical parenchyma cells are visualized with the blue autofluorescence of lignin (340–380 nm excitation, LP 420 emission). (C) Fluorol yellow/acridine orange staining showing suberized epidermis (arrow) and lignified thickenings of cortical parenchyma cells (green). Merged green and red channels (BP 510–530; BP 590–610). (D) Acridine orange staining showing lignified walls (green fluorescence) in xylem and cortex. A combination of transmitted white light and wide-field fluorescence (340–380 nm excitation, LP 420 emission) was used to show the parenchyma cells and the intercellular air spaces in the cortex. (E) The same section as in (D) but visualized only by fluorescence without the transmitted light. Note the blue autofluorescence of Casparian thickenings (arrow) in the endodermal cells that at an early stage of development are not stained with the acridine orange. Bars = 50 µm (A through E).

We use the following protocol of staining with CR and fluorescence microscopy. FAA-fixed sections are firstly washed in running tap water for 30 min. Then, a 0.1 % CR solution in 50 % ethanol is applied for 5–10 min at room temperature. The sections are rinsed two times in distilled water, then placed on a glass slide in a drop of water or aqueous glycerol for observation by fluorescence microscopy. Sections can stay for long time in glycerol without any visible leaking of CR in the background. The optimal excitation/emission of CR is 561 nm/630 nm but CR also can be excited with UV light for simultaneous imaging of blue autofluorescence of lignin and red fluorescence of CR-stained cellulose (Fig. 3B).

**Figure 4.**
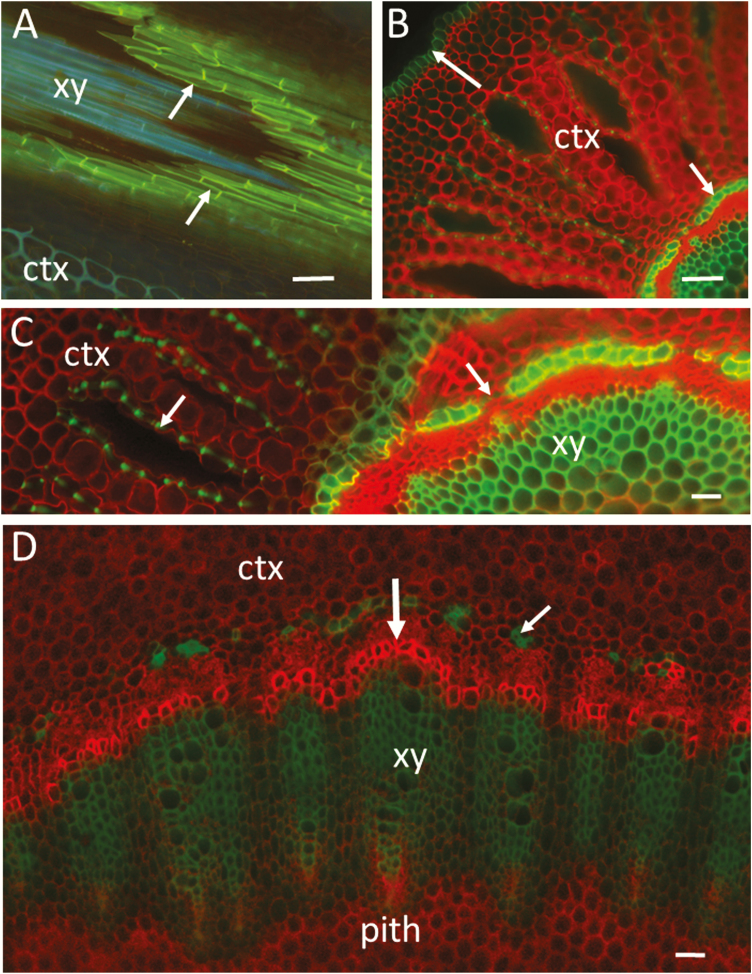
Hand-cut sections of root and stem of a black mangrove plant (*B. gymnorrhiza*) visualized by wide-field fluorescence (Axio Scope A1; Carl Zeiss). (A) Longitudinal-oblique section through the root endodermis (arrows) showing xylem inside the endodermal cylinder. Staining with fluorol yellow (UV excitation and LP 420 emission). Suberized cell walls are yellow (or mixed blue-yellow) and lignified walls (xylem and cortex) emit blue-green autofluorescence. (B) Transverse section of a root showing the cortex, epidermis (upper arrow) and endodermis (lower arrow). Staining with Congo red and two-channel imaging (green ex/em: BP 450–490/BP 500–550, red ex/em: BP 539–563/BP 570–640). The composite image showing red staining of cellulosic walls and green autofluorescence of lignin and suberin. (C) Transverse section of a root showing inner cortex, endodermis, phloem and xylem. Note the green autofluorescence of lignified thickenings of cortical parenchyma cells (left arrow). The right arrow points to a transfusion endodermal cell with no suberin and lignin in the cell wall. The same staining and imaging conditions as in (B). (D) Transverse section through the hypocotyl of the same plant as in (C) showing inner cortex, xylem and small portion of the pith. Developing xylem cells with unlignified walls (vertical arrow) are stained stronger in bright red. The small arrow points to lignified cells on the outer side of the phloem. The same staining and imaging conditions as in (B). Bars = 100 µm (A and B), 50 µm (C and D). ctx, cortex; xy, xylem.

**Figure 5.**
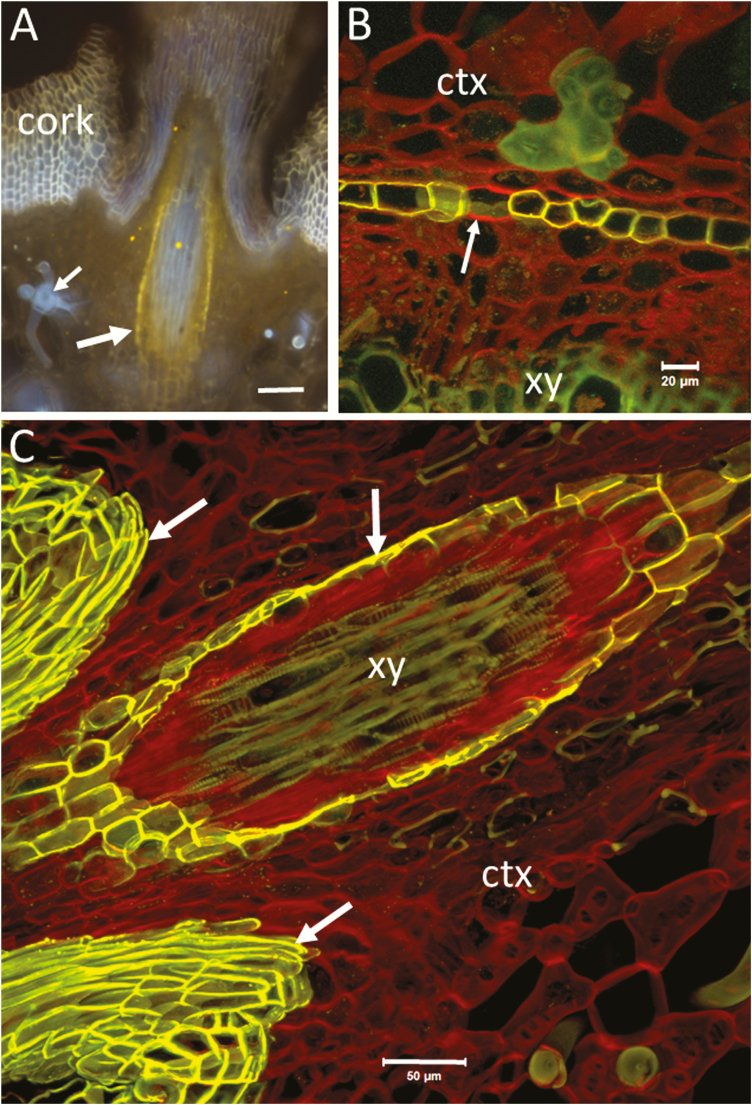
Hand-cut transverse sections of roots of a *Rhizophora apiculata* plant visualized after staining with fluorol yellow (A) and after double staining with fluorol yellow/Congo red (B and C). The image in (A) was obtained by wide-field fluorescence with UV excitation and LP 420 emission (Olympus BX60). The images in (B and C) are merged two channels by CLSM (Zeiss LSM510; ex/em: 458/BP 500–550; 488/LP 560). The green and red emission from fluorol yellow-stained suberized walls is considerably stronger than the green and red from lignified and non-lignified walls, respectively. The resulting image shows red staining of polysaccharides, yellow (overlapped green and red) for suberized cell walls and green for lignin. (A) A lateral rootlet (obliquely cut) shown at the outer region of the cortex of the mother root. The large arrow points to yellow-stained endodermis of the rootlet. Note the blue autofluorescence of xylem inside the endodermal cylinder of the rootlet. The blue and yellow fluorescence of exodermal tissue (cork) indicates presence of lignin and suberin. Blue fluorescence is also emitted from lignified astrosclereids in the cortex (small arrow). (B) A view of the endodermal region by CLSM (maximum projection of 47 optical sections at 0.7-µm intervals; C-Apochromat 40×/1.2 Water). Non-lignified cell walls are stained in red. The arrow points to a transfusion endodermal cell with no suberin or lignin in the cell wall. The autofluorescence of lignified walls of xylem and sclerenchymatic cells in the cortex is shown in green. (C) A similar rootlet as in (A) but visualized in more detail by CLSM (maximum projection of nine optical sections at 2.3-µm intervals; Plan-Apochromat 20×/0.75). The arrows on the left point to exodermis. The vertical arrow points to the endodermis of the rootlet. Note the autofluorescence of xylem inside the endodermal cylinder (green). Phloem and parenchymatic cells with unlignified walls (Congo red staining) occupy the space between the xylem and endodermis. Note also the loose arrangement of cellulosic parenchyma cells in the cortex of the mother root resulting in formation of intercellular air spaces. Bars = 100 µm (A), 20 µm (B) and 50 µm (C). ctx, cortex; xy, xylem.

The images in Fig. 4 were obtained by merging the green (Ex 450–490/EM 500–550) and red (Ex 539–563/EM 570–640) channels. The green channel reveals the autofluorescence of lignified cell walls, which combined with the red signal of CR produces a strong contrast between lignified and primarily cellulosic walls (Fig. 4B–D). Developing xylem cells prior to lignification are stained more strongly with CR than the parenchyma of cortex and pith (Fig. 4D). Probably, the stronger staining with CR is facilitated by the occurrence of various polysaccharides and proteins in the cell walls and cytoplasm of differentiating xylem. Cellulose or other polysaccharide components are also present in the lignified walls; therefore, CR will stain to some extent the lignified walls too (Hubbe *et al.* 2019). Consequently, a single red-channel observation may not be useful for differentiation of lignified and non-lignified walls. It can be seen in Figs 4 and 5 that the contrast between unlignified and lignified walls is effectively enhanced by using the combination of green and red colours (see also Fig. 2C). A greater specificity can be achieved by immunological staining where highly specific interactions between fluorescently labelled antibodies and antigens are used. Immuno-labelling has been applied in studies of complex lignocellulosic materials by many authors (Funada *et al.* 2001; Funada 2002; Donaldson and Knox 2012; see review by Paës *et al.* 2017). Analogous to antibodies, CBDs (cellulose- or carbohydrate-binding domains) or CBMs (cellulose-binding modules), proteins with affinity to specific carbohydrate structures, have been applied for microscopic detection of cellulose (Hildén *et al.* 2003; Daniel *et al.* 2006; Kawakubo *et al.* 2010; Yang *et al.* 2015; Arola and Linder 2016). Novy *et al.* (2019), differentiated between organized and disorganized cellulose through simultaneous use of CBMs labelled with different fluorophores. As hundreds of carbohydrate-binding domains, many already fluorescently labelled, are commercially available, this represents an excellent toolbox for investigating carbohydrate localization, as long as the carbohydrates are accessible to the rather large proteins.

#### Calcofluor staining of cellulose and red autofluorescence of lignin.

Calcofluor white M2R (molecular weight 917) has been used as a fluorescent brightener in the paper and textile industry, as well as for studying cell walls of fungi, bacteria and plants (Hughes and McCully 1975; Galbraith 1981; Wood *et al.* 1983; Harrington and Hageage 2003; Hoch *et al.* 2005). When excited with UV light or 405 laser, calcofluor white produces blue fluorescence of cellulosic walls (Donaldson and Bond 2005; Kitin *et al.* 2010). The protocol by Nakaba *et al.* (2015) for staining cambium with calcofluor white is suitable for differentiation of cellulosic or chitinous walls from the autofluorescence of lignified or suberized portions of cell walls (Fig. 2). Figure 2A shows the unlignified walls of gelatinous fibres, ray cells and tyloses in blue, in contrast to the red autofluorescence of the lignified wall of a vessel element. Furthermore, the confocal image in Fig. 2A shows intervessel bordered pits in the lignified vessel wall (large arrow) in contrast to parenchyma-to-vessel contact pits with large unlignified membranes (small arrows on the right side of the image). The half-bordered contact pits in Fig. 2A are similar to those that are indicated with arrows in Fig. 2B. The formation of parenchyma-to-vessel contact pits in poplar was studied by Murakami *et al.* (1999).

We use the following protocol of staining and fluorescence microscopy. The calcofluor staining procedure should start with washing fixed sections in running tap water for 15–30 min. Then, stain with a 0.01 % wt/vol aqueous calcofluor white M2R (syn. fluorescent brightener 28) for 5 min at room temperature and protect from light. Adding few drops of 1 N NaOH is recommended (Ruzin *et al.* 1999). Rinse the sections with distilled water after the staining. Thick sections should be rinsed at least 10 times for removing the excess calcofluor. Temporary microscope slides can be prepared by mounting in water or glycerol. Observe with excitation of 340–380 nm and long-pass emission LP 420, or by dual-channel imaging using blue (DAPI) and red (Texas Red) filter combinations (Fig. 2A). Note that calcofluor is often sold in combination with a small amount of blue dye (often Evans blue) to quench unwanted background fluorescence.

#### Calcofluor/safranin for differentiation of cellulose and lignin domains.

Safranin (molecular weight 351) is an inexpensive, general stain for histological sections and is available in most research laboratories. It has a long history of applications and continues to be popular today for studies of plant cellular structure and for visualizing xylem cells (Kitin *et al.* 2000; Bond *et al.* 2008; Rahman *et al.* 2019). Sections can be quickly stained with 0.1 % aqueous safranin for 3–5 min at room temperature. Differentiation between lignified (stained) and non-lignified (unstained) cells can be achieved by rinsing in an aqueous ethanol series as the following: (25, 50, 70 and 95 % ethanol) for 30 min with several washes at each concentration until no leach of dye is visible. Because safranin will continue to leach out from cell walls if the sections remain in ethanol, return to water through an ethanol series (70, 50 and 25 % ethanol) for 15–30 min at each concentration. The time-consuming procedure with gradual ethanol series is aimed to preserve the morphology of subcellular structures and has been adapted from transmission electron microscopy (TEM) protocols. It allows differentiating cell walls or protoplasmic content to be studied by correlative light and electron microscopy. Moreover, the continuous rinsing in ethanol solutions will clear thick sections not only from the excess dye but also from some pigments and extractives which will improve the clarity of cell wall observations. Then follow the calcofluor staining procedure as described earlier.

We use the following protocol of staining and fluorescence microscopy. Sections stained with safranin can be immersed in water or immersion oil for preparation of temporary microscope slides (Donaldson and Bond 2005; Bond *et al.* 2008). Sections can be also observed in aqueous glycerol; however, they must be rinsed in fresh aqueous glycerol immediately before observation because safranin tends to leach out and produce fluorescent background in glycerol mounting medium. Observe by wide-field fluorescence microscopy with 340–380 nm excitation and a LP emission filter (LP 420) or by CLSM with a dual-channel excitation/emission for calcofluor 405 nm/410–485 nm, and for safranin 488 nm/515–565 nm or 541 nm/LP 590 nm (Nakaba *et al.* 2015). The results are blue fluorescence from cellulosic portions of the walls and green or red fluorescence from lignified walls. The calcofluor signal in Fig. 2B shows exposed cellulose in pit membranes or in artefactual cracks in the cell walls caused during drying or sectioning of the wood. In Fig. 2C, green colour was artificially assigned to the calcofluor fluorescent channel for a better contrast with the red fluorescence from lignin. The exposed cellulose in the xylem cell walls in Fig. 2C is caused by a white rot fungal degradation. The calcofluor signal is stronger in the earlywood (ew) which indicates that the white rot fungus degrades lignin faster in the ew than in the latewood (lw).

Fluorophores with affinity to lignin have been studied by many researchers (Donaldson and Bond 2005; Drnovšek and Perdih 2005; Bond *et al.* 2008; Chabbert *et al.* 2018; Hubbe *et al.* 2019). The intensity of the safranin signal varies in relation to the amount of lignin (Drnovšek and Perdih 2005; Bond *et al.* 2008). Depending on the concentration of binding sites, interaction between dye molecules may lead to shifts in the intensity or spectrum of fluorescence due to quenching or FRET. Therefore, various degrees of lignification in different cell types and cell wall layers can be qualitatively visualized with safranin staining (Fig. 1A). Similarly, the autofluorescence of conifer xylem cells is stronger in the middle lamella and cell corners where the lignin proportion is higher (Donaldson 2013). Besides safranin, other green or red fluorescent dyes that can produce contrast to calcofluor-stained cellulosic walls include AO, acriflavine, basic fuchsin and crystal violet (Donaldson *et al.* 2001; Donaldson 2002; Donaldson and Bond 2005; Drnovšek and Perdih 2005; Kapp *et al.* 2015; Houtman *et al.* 2016; Zhang *et al.* 2017). By contrast, acidic (negatively charged) dyes, such as acid fuchsin, tend not to stick to lignified cell walls, in our experience. The electrochemical binding between the cationic dye AO and cell wall has been studied stoichiometrically for analysis of the chemical changes during fungal biodegradation of wood (Houtman *et al.* 2016). Acridine orange emits green as a single molecule and red as a dimer. Therefore, when the metachromatic properties are desired, such as staining acid groups on biomass, it has been applied at a concentration where both monomers and dimers were stable (Houtman *et al.* 2016). Others have achieved the same result by putting AO on at high concentration, then extensively rinsing in aqueous ethanol so only the most stable dimers remained (Nakaba *et al.* 2015). The micrographs in Fig. 3C–E show the green fluorescence of lignified walls in AO-stained sections of mangrove roots. Similarly to safranin, AO may stain non-lignified walls either by intercalation (green fluorescence) or by bonding with negatively charged carboxylic functional groups (red fluorescent dimers) (Houtman *et al.* 2016). Nevertheless, clear differentiation between lignified and cellulosic portions of cell walls is achieved because of the considerably weaker fluorescence of safranin-, or AO-stained polysaccharides, relative to the blue calcofluor signal (Fig. 2B and C). For more discussion on the mechanisms of staining involving two dyes for colour differentiation of lignin domains in cellulosic walls, see Hubbe *et al.* (2019).

Combining different imaging techniques may reveal important details. For example, in Fig. 3B, the CR staining shows the cellulosic walls of phloem and parenchyma in the vascular tissue and cortex, which are not visible (or poorly visible) without CR staining (Fig. 3A and C). The parenchyma of the cortex and intercellular spaces can be also seen in combined fluorescence and transmitted-light images (Fig. 3D). However, the Casparian thickenings of the endodermis are not visible in Fig. 3D, while they can be clearly seen in the single fluorescence image of the same section (Fig. 3E). The fact that the Casparian thickenings are not stained with AO suggests that the lignocellulosic complex in the endodermal cell wall is different from those in the xylem and cortical cells, at least in this stage of root development (Fig. 3E).

#### Fluorescence staining for suberin and lipids.

A large number of fluorescent probes for lipids and cell membranes have been developed mainly for medical research as lipophilicity is an important property of drugs. Berberine hemisulfate (BH) and FY are commonly used fluorescent stains for lipids and suberin in the traditional plant anatomy (Brundrett *et al.* 1991; Lux *et al.* 2005). Sudan IV can fluorescently stain suberin with excitation/emission 561 nm/600–700 nm which was used to differentiate suberin from lignin (Donaldson *et al.* 2015). Nile red has been introduced as a lipophilic dye by King (1947) and Greenspan *et al.* (1985), and recently Nile red has been selected for staining of endodermis in *Arabidopsis* roots because of the compatibility of this dye with the tissue-clearing medium ‘ClearSee’ (Ursache *et al.* 2018). However, Brundrett *et al.* (1991) noted some unspecific staining with Nile red of cell walls containing phenolic substances. The specificity of the Nile red dye to different ontogenetic stages of endodermal development remains relatively unexplored. On the other hand, techniques of fluorescent staining with FY described by Brundrett *et al.* (1988, 1991) have been followed by many researchers in studies of exodermis and endodermis of roots, or suberized cell layers (Lux *et al.* 2005; Cai *et al.* 2011; Martinka *et al.* 2012; Zelko *et al.* 2012; Krishnamurthy *et al.* 2014; Soukup and Tylová 2014; Rossi *et al.* 2017). Different stages of endodermal and exodermal development can be distinguished by staining with BH and FY. Berberine hemisulfate staining followed by aniline blue counter staining and UV fluorescence microscopy highlights the Casparian strips (Brundrett *et al.* 1988; Kreszies *et al.* 2018). Accordingly, the Casparian strips in *Arabidopsis* contained only lignin polymer without suberin in early stage of development (Naseer *et al.* 2012). The deposition of lamellar suberin in endodermal walls, which may occur simultaneously or after the deposition of lignin, can be visualized with FY staining (Brundett *et al.* 1988, 1991; Lux *et al.* 2005; Song *et al.* 2019).

Fluorol yellow 088 (synonyms: 2,8-dimethylnaphtho[3,2,1-kl]xanthene; Solvent Green 4, CAS 81-37-8) is not charged and so has very low water solubility. The absorption/emission characteristic of FY is λex\λem (MeOH) = 450/515 nm. Below, we discuss popular protocols for staining with FY (molecular weight 296) and demonstrate our experience of multichannel labelling of cellulosic, lignified and suberized portions of cell walls.

One of the main challenges of using FY has been the establishment of an appropriate solvent that allows sufficient infiltration and staining of plant tissues and fatty substances. The FY dye is strongly soluble in dimethyl sulfoxide, dimethylformamide, acetonitrile, ethyl acetate and chloroform and poorly soluble in water. Previously used solvents of lipid stains include aqueous alcohols such as ethanol or isopropanol diluted in water, ethanol-acetone-water, as well as ethylene glycol or propylene glycol (Gutiérrez and Lillie 1965; see also Brundrett *et al.* 1991). However, alcohols may extract lipids or cause unwanted destaining effects in plant tissues. In addition, dye precipitation may occur when the staining solution is mixed with water in hydrated (fresh) plant material. To reduce volatility of the solvent and resolve problems with dye precipitation, glycol, dextran or PEG have been added to the solvent mixtures (Catalano and Lillie 1975; O’Brien and McCully 1981; Brundrett *et al.* 1991). Still remaining technical problem was to balance the non-polar properties of the solvent required to keep the lipid dye in solution and at the same time to allow strong staining. According to Brundrett *et al.* (1991), efficient staining of lipids occurs by partitioning (absorption) of the dye into hydrophobic domains of the cells. Therefore, lower solubility of FY in the staining solution should facilitate staining of lipids in plant tissues. Brundrett *et al.* (1991) suggested PEG 400 mixed with aqueous glycerol as an effective carrier of the FY dye (PEG 400/glycerol/water = 10/9/1, vol/vol). The maximum solubility of FY in PEG 400-glycerol was measured to be 0.1 %, in pure PEG 400 was 1 % and in various fatty substances, such as terpenoid or sterol, was ≥3 % (Brundrett *et al.* 1991). It was demonstrated that PEG 400 efficiently dissolved FY and when mixed with aqueous glycerol allowed intense and specific staining of lipids and suberin lamellae. When pure PEG 400 was used as a solvent, staining was weak presumably because PEG retains the dye rather than allowing it to selectively stain fatty cellular substances. Lux *et al.* (2005, 2015) further developed the procedure for staining suberin and Casparian strips in roots. Instead of PEG 400, they used lactic acid as the solvent of FY or BH which resulted in strong staining. Furthermore, they cleared hand sections and whole-mount root samples in lactic acid saturated with chloral hydrate claiming it provided superior imaging of suberized cell layers.

Alternatively, we further simplify the suberin-staining procedure by using glycerol as both the FY solvent and the clearing medium. The solubility of FY in pure glycerol is 0.1 %, similar to that in the PEG-glycerol solvent. Therefore, glycerol, as well as aqueous glycerol, act as excellent carriers of FY for staining plant lipids. The following protocol allowed for quick and high-quality observations of suberized cell layers in thick sections of plant roots and stems.

#### Fluorol yellow in glycerol for labelling of suberin and lipids.

FAA-fixed plant material must be rinsed for 15–30 min in running water while WEG-fixed samples do not need to be washed. Then change the storage solution to 50 % glycerol for 1 h.

Immerse the sample in a staining solution of 0.05 % FY in glycerol for 1 h (15-µm-thick sections), overnight (40-µm-thick sections) or at 60 °C overnight (thicker than 40 µm slices or whole mounts). The sections can be stored for few days in a refrigerator in dark without noticeable fading of the stained tissues. Before observation, change the staining solution with fresh glycerol (if any FY precipitation is detectable under the microscope, continue rinsing the stained sample in glycerol until the tissue is well cleared). Many clearing agents, such as potassium hydroxide and sodium hypochlorite, chemically degrade some fractions of the cell wall. The pure glycerol (refractive index 1.47) impregnates relatively non-destructively any kind of plant cell wall and serves as a clearing agent with excellent optical properties. To achieve good clearing of larger samples that contain lignified, suberized or cutinized tissues, we keep in glycerol overnight.

Mount in glycerol on microscope slides and use a standard FITC filter combination or 488 excitation and a BP 510–590. Fluorol yellow is also excited with UV light providing the possibility for a simultaneous imaging of lignin autofluorescence and the FY signal (Fig. 5A). The results are strong FY staining of suberized cell walls with no- or negligible precipitation of FY. Cork and suberized endodermis can be clearly observed in young portions of roots as well is in secondary thickened roots and stems (Figs 3A–C, 4A and 5). Ursache *et al.* (2018) reported that FY staining was not possible to use in association with the tissue-clearing medium ClearSee. We found that glycerol served as an excellent medium for FY staining of fatty substances as well as for clearing of the tissue sections, providing high-quality microscopic images. Furthermore, glycerol is inexpensive, easy to handle and might be particularly appropriate for use in secondary xylem studies as it can easily penetrate and clear lignified tissues.

#### Double staining with FY/CR for differentiation of suberized, lignified or cellulosic portions of cell walls.

The strong fluorescence and yellow colour of FY when bound to suberin makes it suitable for multicolour imaging in combination with popular blue, green or red dyes. FAA-fixed sections are firstly rinsed in water as described above. Then immerse in a 0.1 % solution of CR in 50 % ethanol for 15–30 min at room temperature. Rinse the stained sections in glycerol solutions in the following sequence: 10 min in glycerol/ethanol/water, 1/1/1 (vol/vol); 20 min in 90 % aqueous glycerol. Follow with the FY staining steps as described above.

**Figure 6.**
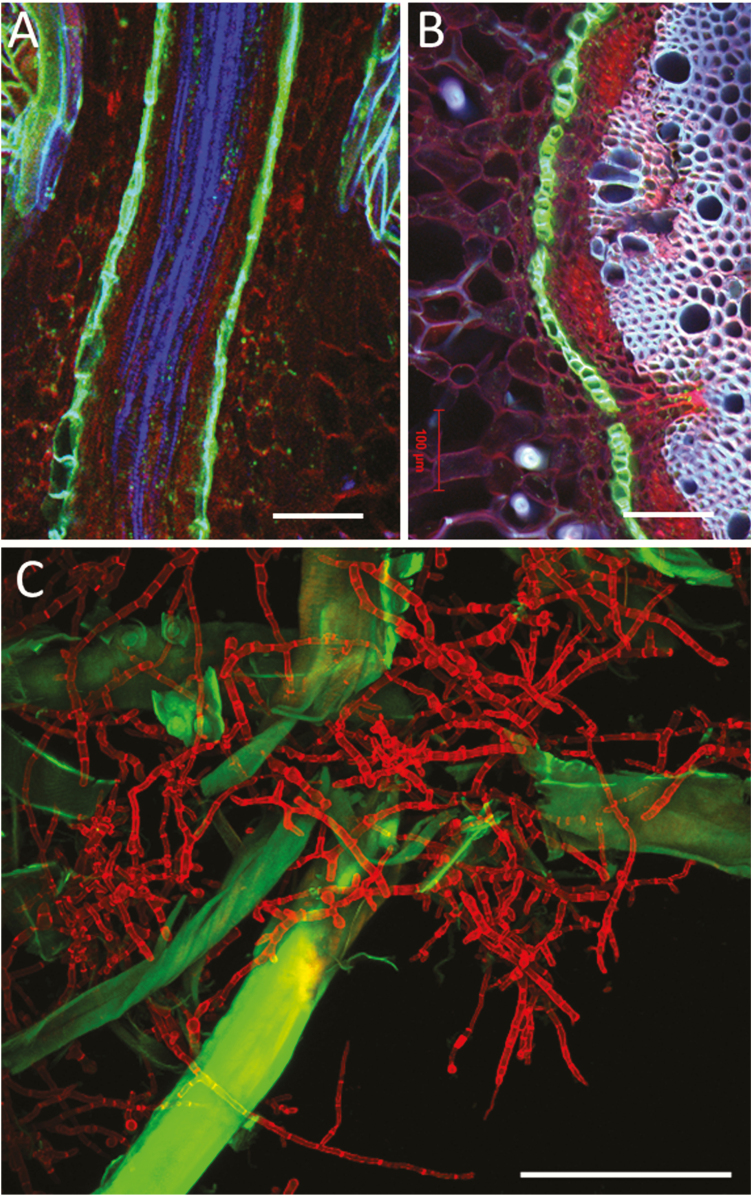
Multichannel imaging by CLSM. Hand-cut longitudinal (A) and transverse (B) sections of a root of *Rhizophora apiculata* visualized after double staining with fluorol yellow/Congo red. Single optical sections by CLSM (Zeiss LSM 880, Axio Imager 2, Objective lens: Plan-Apochromat 10/0.45 M27). Merged three channels (ex/em of 405/BP 415–498; 488/BP 543–579; 561/BP 590–630) showing blue for lignin, green for suberized cell walls and red staining of polysaccharides. (C) Maximum image projection of *Tricoderma reesei* hypha (false colour red) with oxidized lignin-bearing softwood fibres (green). Red hypha (calcofluor) collected with ex/em 405/424–502, green emission from FM 5-95 (fibre) ex/em 488/499–591 (Zeiss LSM 710). The FM 5-95 dye adsorbed on oxidized lignin producing much stronger signal than in membranes. Lignin autofluorescence has insignificant contribution. Bars = 100 µm.

Place sections on a glass slide, apply a drop of glycerol and mount the sections with coverslips. For simultaneous imaging of FY and CR staining, observe by wide-field fluorescence using UV illumination and a long-pass emission (e.g. 365 nm peak excitation; and barrier filter LP 420). An example of a wide-field fluorescence image showing lignified, suberized and cellulosic walls is provided in Fig. 3B. For comparison, Figs 3A and C and 5A show FY staining and autofluorescence without CR. For two-channel imaging, use the FITC filter set for the FY channel, and the Texas Red set or 561/LP 590 for CR. By spectral imaging, we found that the fluorescence of FY-stained suberized walls in the range of 510–630 nm is considerably stronger than the native fluorescence of lignin and suberin. Therefore, FY-stained walls can be easily observed in the FITC channel. Furthermore, the FY-stained cell walls, when excited with a blue laser, fluoresce considerably stronger than the CR-stained non-lignified walls. This provides a good possibility for distinction between suberized, lignified and non-lignified walls with a single Argon laser and dual red and green channel imaging. The merged red/green images in Fig. 5B and C show clear differentiation between the FY-stained suberized cell walls (the mixture of strong green and red signals results in yellow), the CR-stained non-lignified walls in red and the green autofluorescence of lignified walls. The standard DAPI channel when available can be also added for imaging the autofluorescence of lignified cell walls in blue (Fig. 6A and B).

## Conclusions

Microtome- or hand-cut sections and planed surfaces can be prepared relatively easy and quickly from either chemically fixed or frozen plant specimens. The autofluorescence of various plant substances can be extremely useful in plant anatomical and histochemical studies, in particular for cryo-microscopy where histochemical staining might be difficult to achieve. Future plant anatomical and histochemical studies will benefit from knowledge on the spectra of natural fluorescence of the variety of plant cell types and cell wall components.

Calcofluor white staining (blue) and autofluorescence of lignin and suberin (red) are useful for differentiation of cellulosic, lignified and suberized portions of cell walls. Adding safranin or AO staining results in stronger fluorescence of lignin and more contrast between lignified and non-lignified walls compared with autofluorescence. However, safranin and AO are not strongly specific to lignin and, depending on the acidic environment, these dyes can also bind to hemicellulose, cellulose or proteins. Therefore, single green or red channel imaging of safranin- or AO-stained lignocellulosic cells should be interpreted with care. By contrast, imaging with a counterstain such as calcoflour can sometimes dramatically improve the ability to distinguish tissue types.

Blue or green autofluorescence of lignin in combination with CR-stained cellulose, and FY staining of suberin can clearly differentiate between lignified, suberized and non-lignified cell walls in root and stem tissues. Glycerol can serve as the carrier of FY for staining of suberin and lipids. The absence of background fluorescence and precipitates in the microscopic preparations shows that glycerol is a suitable mounting medium for both CR- and FY-stained plant material. At the same time, glycerol serves as an effective and non-destructive clearing medium for primary and secondary root and stem tissues allowing for clear observation of thick histological preparations by wide-field fluorescence microscopy or CLSM.

The WEG mixture is an excellent preservative and clearing agent which has been overlooked and rarely used in plant anatomical research. The presented protocols for sample preparation and multichannel imaging are simple and rapid while providing high-quality 3D imaging of all cellular structures together with chemical information on the distribution of lignin and suberin in cellulosic walls.
